# Large language models versus expert clinicians in optimizing Chinese patient education materials for temporomandibular disorders: a mixed-methods study integrating readability, accuracy, actionability, and cultural adaptability

**DOI:** 10.3389/fpubh.2026.1923536

**Published:** 2026-08-04

**Authors:** Jing Han, Yan-li Shi, Li Dai

**Affiliations:** 1 Department of Stomatology, Jinan Maternal and Child Health Care Hospital, Jinan, China; 2 Department of Stomatology, Shandong Provincial Hospital Affiliated to Shandong First Medical University, Jinan, China

**Keywords:** actionability, Chinese, cultural adaptability, human-AI collaboration, large language models, patient education, temporomandibular disorders

## Abstract

**Objective:**

To evaluate the performance of large language models (LLMs) and expert clinicians in optimizing Chinese patient education materials (PEMs) for temporomandibular disorders (TMD) across readability, accuracy, actionability, and cultural adaptability, and to determine whether a human-AI collaboration model can achieve an optimal balance among these dimensions.

**Methods:**

Fifteen TMD education topics were selected through a Delphi consensus process. Four groups of PEMs were generated: original texts (Group A), LLM-rewritten texts (Group B, Claude Opus 4.6), clinician-rewritten texts (Group C), and human-AI collaboration texts (Group D). Blinded assessments were conducted by a professional panel (*n* = 3) and a health literacy-stratified patient panel (*n* = 6). Readability was measured by sentence length and common character proportion, accuracy by 5-point expert ratings against standardized checklists, actionability by the PEMAT instrument, and cultural adaptability by qualitative thematic analysis. Prompt sensitivity was tested across three distinct styles.

**Results:**

LLM-rewritten texts demonstrated significantly shorter sentences (21.1 vs. 33.9 characters, *p* < 0.001) and substantially higher actionability (95.6% vs. 16.7%, *p* < 0.001) compared with original texts. Clinician-rewritten texts achieved the highest accuracy (4.48 vs. 3.23, *p* < 0.001) but low actionability (46.7%). The human-AI collaboration model matched clinician accuracy (4.49, *p* = 1.000) while preserving LLM actionability (95.6%), with 72% less clinician time. Sensitivity analysis confirmed actionability robustness under empathetic and authoritative prompts but attenuation under a concise prompt. Qualitative analysis identified four cultural adaptability themes, with LLMs excelling in terminology localization and behavioral structuring but showing limitations in TCM conceptual depth.

**Conclusion:**

The human-AI collaboration model represents a promising and balanced approach for producing Chinese TMD patient education materials, combining LLM strengths in structural optimization with expert clinician oversight for accuracy and cultural appropriateness.

## 1 Introduction

Temporomandibular disorders (TMD) affect 5–15% of the general population, with a marked female predominance, and represent one of the most common sources of chronic orofacial pain (1, 2). High-quality patient education is a first-line, foundational component of TMD management (3, 4), yet its delivery is undermined by a persistent structural barrier: the average outpatient consultation provides less than 20 min for history-taking, examination, diagnosis, and treatment initiation (5). Consequently, patient education relies heavily on written materials—the effectiveness of which depends entirely on their quality.

A substantial body of evidence has documented that patient education materials (PEMs) across medical specialties consistently exceed recommended readability thresholds (6, 7). Beyond readability, the construct of actionability—the degree to which a material enables readers to identify concrete actions they can take—captures an equally critical dimension of PEM quality (8). Even materials with acceptable readability frequently fall short on actionability, describing problems without specifying step-by-step solutions (9). In TMD, where effective self-management demands specific behaviors—applying heat or cold with correct timing, performing jaw exercises with proper technique, adhering to dietary modifications—this deficit carries direct clinical consequences.

The Chinese-language context compounds these challenges. Chinese medical terminology is characterized by high-density compound characters lacking intuitive vernacular equivalents, and only approximately 25% of Chinese adults possess adequate health literacy (10, 11). Cultural factors—including dietary practices, communication norms, and the integration of Traditional Chinese Medicine (TCM) into mainstream healthcare—further shape how patients understand and act upon health information. Despite this, no study has systematically evaluated Chinese-language TMD PEMs across multiple quality dimensions or assessed their cultural adaptability.

Large language models (LLMs) have opened a new frontier in health communication, offering the capacity to instantaneously rewrite complex medical information at specified reading levels and in specified languages (12, 13). Recent studies across multiple specialties have consistently found that LLM-generated PEMs demonstrate superior readability compared with original materials (14–16). Comparative evaluations have further shown that this advantage is shared across contemporary LLMs, including ChatGPT-5 and DeepSeek V3, which produce patient education materials of comparable clinical utility (17). However, the accuracy of LLM-generated content remains “generally correct but incompletely reliable,” with performance varying across clinical domains and falling below thresholds required for unsupervised application (17, 18). A small number of studies have explored collaborative workflows in which LLMs produce first drafts subsequently reviewed by content experts, demonstrating that this approach can capture AI’s readability advantages while maintaining expert-level accuracy (19).

The present study addresses these gaps through four unique contributions: (1) the first evaluation of LLMs for Chinese-language TMD patient education, with explicit attention to cultural adaptability and TCM integration; (2) the first integration of actionability and cultural adaptability alongside readability and accuracy in a single evaluation framework; (3) the first systematic comparison of four major LLMs (GPT-4, Claude Opus 4.6, Gemini 3.0 Pro, and DeepSeek-V2) for patient education in a non-English, culturally specific context; and (4) an empirical evaluation of a human-AI collaboration workflow with quantified time efficiency gains, moving beyond AI-versus-human comparisons to a clinically implementable model. To ensure real-world relevance, we recruited TMD patients stratified by health literacy level as assessors, and employed a three-stage Delphi consensus process to select 15 core educational topics spanning Western medical content and TCM.

## 2 Methods

### 2.1 Study design

This cross-sectional, mixed-methods study evaluated Chinese-language patient education materials (PEMs) for temporomandibular disorders (TMD) generated through four approaches, integrating quantitative assessments of readability, accuracy, and actionability with qualitative thematic analysis of cultural adaptability.

### 2.2 Topic selection: three-stage Delphi consensus process

#### 2.2.1 Stage 1: initial topic pool construction

An initial pool of 35 candidate topics was compiled from four sources: the DC/TMD diagnostic criteria (20); the AAOP Clinical Practice Guidelines (6th edition) (3); consensus statements of the Chinese Stomatological Association TMD Committee (2019–2024); and a PubMed search of systematic reviews on TMD patient education (2020–2025). Topics were organized into six domains: disease knowledge (*n* = 6), etiology and risk factors (*n* = 5), diagnosis and examination (*n* = 4), treatment modalities (*n* = 7), self-management and rehabilitation (*n* = 8), and pharmacotherapy, TCM, and integrative management (*n* = 5).

#### 2.2.2 Stage 2: two-round Delphi consensus

A five-member multidisciplinary panel was convened: a TMD specialist, an oral and maxillofacial surgeon, a general dental practitioner, a rehabilitation medicine specialist, and a nursing/patient education specialist. In round one, each expert independently rated all 35 topics on clinical importance and patient concern frequency using a 5-point Likert scale. A composite score was calculated as (clinical importance × 0.6) + (patient concern frequency × 0.4). In round two, experts convened online to discuss topics with high inter-expert variability (CV > 0.25) and newly proposed topics, followed by anonymous real-time voting. Topics achieving ≥60% agreement were retained.

#### 2.2.3 Stage 3: final confirmation

The 15 highest-scoring topics were reviewed by an independent senior TMD specialist to confirm no critical omissions. The final topics spanned Western medical content (etiology, imaging, conservative treatment, occlusal splint therapy, surgical indications, pharmacotherapy) and TCM content (acupuncture). The complete list appears in Supplementary Table S1.

### 2.3 Generation of patient education materials

Four groups of PEMs were generated for each of the 15 topics.

#### 2.3.1 Group A: original texts (baseline)

Original PEMs were derived from three sources: existing clinical handouts from the Department of Oral and Maxillofacial Surgery, Shandong Provincial Hospital (~50%); guideline excerpts from AAOP and Chinese Stomatological Association consensus statements (~30%); and researcher-drafted texts for topics lacking existing materials, written prior to expert review (~20%). All texts were standardized to 300–500 Chinese characters, maintained university-level reading difficulty, used professional terminology without lay explanations, and presented instructions as general principles. Medical accuracy was independently reviewed by a TMD specialist and a TCM specialist not involved in subsequent phases.

#### 2.3.2 Group B: LLM-rewritten texts

Each Group A text was input into four LLMs: GPT-4 (OpenAI, USA), Claude Opus 4.6 (Anthropic, USA), Gemini 3.0 Pro (Google, USA), and DeepSeek-V2 (DeepSeek-AI, China). A uniform prompt (Supplementary Appendix A) instructed models to rewrite texts for Chinese adults at a sixth-grade reading level, using warm and empathetic language, culturally appropriate examples, and step-by-step actionable instructions while maintaining medical accuracy. For TCM content, the prompt required accurate concept presentation and emphasis on qualified professional guidance. All models were accessed via their standard web interfaces (ChatGPT Plus, claude.ai, Google AI Studio, chat.deepseek.com) with default temperature settings. Generations were completed between January and May 2026. Outputs were designated B1 (GPT-4), B2 (Claude Opus 4.6), B3 (Gemini 3.0 Pro), and B4 (DeepSeek-V2).

#### 2.3.3 Group C: expert clinician-rewritten texts

Two TMD specialists (>10 years’ experience, not involved in Delphi, Group A authorship, or assessments) rewrote each Group A text to produce what they considered optimal for patient education, without constraints on time, style, or approach. Texts were collected after one week.

#### 2.3.4 Group D: human-AI collaboration texts

The optimal LLM was selected based on combined accuracy and actionability performance. Claude Opus 4.6 achieved the highest accuracy (3.23 ± 0.69) among the four LLMs with actionability (95.6% ± 11.3%) equivalent to the best-performing models. Its outputs were provided to the same two clinicians, who reviewed and revised the AI-generated drafts for medical accuracy, clinical appropriateness, and cultural suitability while preserving effective structural and language features.

The main four-group analysis included 60 texts (15 topics × 4 groups: A, B-Claude Opus 4.6, C, D). Additional LLM subgroups (GPT-4, Gemini 3.0 pro, DeepSeek-V2) and sensitivity analysis texts were analyzed separately.

### 2.4 Prompt sensitivity analysis

An exploratory sensitivity analysis tested three prompt styles on three topics selected for high actionability requirements (Topic 11: dos and don’ts; Topic 12: acupuncture; Topic 15: occlusal splint therapy). Claude Opus 4.6 was tested with: Prompt A (original, empathetic), Prompt B (concise and objective, without emotional language), and Prompt C (authoritative, adopting a senior clinician’s persona). Full prompts appear in Supplementary Appendix A. Outputs were assessed using the same PEMAT and accuracy instruments as the main experiment.

### 2.5 Text preprocessing

All texts were manually cleaned to retain patient-directed content only. Formatting artifacts, extraneous line breaks, and conversational framing were removed. Titles and headings were retained when they contributed to organization. Texts were anonymized, assigned random codes, and compiled into assessment booklets with randomized presentation order within each topic.

### 2.6 Blinded assessment procedures

Four independent evaluation streams were employed. All assessors were blinded to text source.

#### 2.6.1 Objective readability

Readability was assessed using mean sentence length and common character proportion. Sentences were delimited by periods (。), question marks (?), exclamation marks (!), and semicolons (;); standalone titles were excluded from sentence counts. Common character proportion was calculated as occurrences of characters from the Grade-1 Commonly Used Standard Chinese Characters list (State Language Commission of China, 2013; 3,500 most frequent characters) divided by total character occurrences, excluding punctuation, Arabic numerals, and Latin letters. Professional term density was computed as 100% − common character proportion. Counts were obtained using Microsoft Word; character classification was performed via Python (v3.11) script.

#### 2.6.2 Professional expert review

Three independent clinicians (one general dental practitioner, one oral and maxillofacial surgeon, one TMD specialist) rated accuracy and completeness on a 5-point Likert scale (1 = multiple critical errors/omissions, 5 = fully accurate and complete) using a pre-established checklist based on DC/TMD criteria (20) and AAOP guidelines (3) (Supplementary Appendix B).

#### 2.6.3 Educational quality assessment

Actionability was assessed using the PEMAT-P actionability subscale (Chinese version) (8). Each item was rated as Agree (1), Disagree (0), or Not Applicable (excluded). Scores were calculated as (total points /total applicable items) × 100%, with ≥70% indicating acceptable actionability. Two trained raters independently scored all texts; discrepancies were resolved through discussion.

#### 2.6.4 Patient-based evaluation

Six adults with confirmed TMD were recruited. Inclusion criteria: age ≥18 years, diagnosis confirmed ≥1 month prior, native Chinese speaker, basic reading ability, written informed consent. Exclusion criteria: healthcare professional/student, severe cognitive/psychiatric impairment, uncorrected visual impairment. Health literacy was assessed using the Chinese version of the Newest Vital Sign (NVS) (21); patients were stratified into low (0–2), moderate (3–4), and high (5–6) levels, with two per stratum. Each patient evaluated a randomly assigned subset of 20–24 texts and rated perceived ease of understanding on a 5-point Likert scale.

### 2.7 Cultural adaptability: qualitative thematic analysis

Two researchers independently coded a 20% random sample of all 90 texts using a framework with four deductive dimensions (terminology localization, dietary/lifestyle recommendation concordance, cultural register of empathy, TCM content safety and specificity) and permitting inductive codes (22). Inter-coder agreement for the 20% double-coded sample was calculated as the proportion of text segments where both coders assigned the same primary code. Agreement exceeded 80% for all four predefined dimensions. Discrepancies in coding were resolved through iterative discussion between the two coders. When consensus could not be reached on a specific segment, a third researcher with expertise in cross-cultural health communication served as an arbiter. Following resolution of discrepancies, the coding framework was refined, and one coder applied the finalized framework to the remaining 80% of texts. Codes were organized into themes through constant comparison until conceptual saturation. Representative quotations were translated by bilingual team members with back-translation verification.

### 2.8 Efficiency assessment

Generation time was recorded for each method: automatically for Group B, and via self-reported time logs for Groups C and D. Mean times per text were compared across groups.

### 2.9 Reviewer training and interrater agreement

All reviewers underwent calibration using pilot texts not included in the final analysis. Interrater agreement was assessed using ICC (two-way random-effects model, absolute agreement) for accuracy and PEMAT ratings, interpreted per Koo and Li (23). Disagreements were resolved through consensus discussion.

### 2.10 Statistical analysis

Analyses were performed using SPSS v27.0. Continuous variables were expressed as mean ± SD; normality was assessed via Shapiro–Wilk test. Repeated-measures ANOVA with Bonferroni correction was used for normally distributed variables; the Friedman test with Dunn’s post-hoc test was used for non-normally distributed data. For the sensitivity analysis, descriptive comparisons were presented for PEMAT scores given the small sample (*n* = 3 per condition); accuracy scores were analyzed via Friedman test with Dunn’s post-hoc test. A two-sided *p* < 0.05 was considered significant.

For comparisons across the four groups (Group A, Group B, Group C, Group D), repeated-measures analysis of variance (ANOVA) with Bonferroni correction for post-hoc pairwise comparisons were used for normally distributed variables. For non-normally distributed variables, the Friedman test with Dunn’s post-hoc test was employed. For the prompt sensitivity analysis, given the small number of topics (*n* = 3 per condition), descriptive comparisons were presented for PEMAT scores; for accuracy scores, the Friedman test with Dunn’s post-hoc test was used.

A two-sided *p* value < 0.05 was considered statistically significant.

## 3 Results

### 3.1 Participant characteristics

Five experts participated in the Delphi consensus process (4 female, 1 male; mean age: 41.8 ± 7.3 years; range: 33–52), with backgrounds spanning TMD surgery (*n* = 1), oral and maxillofacial surgery (*n* = 1), general dentistry (*n* = 1), rehabilitation medicine (*n* = 1), and nursing/patient education (*n* = 1). Mean clinical experience was 16.2 ± 8.6 years (range: 5–26). The two-round Delphi process yielded consensus on 15 core educational topics from 35 initial candidates.

The professional assessment panel comprised three independent clinicians (1 female, 2 male; mean age: 39.0 ± 4.6 years; range: 34–43): one general dental practitioner, one oral and maxillofacial surgeon, and one TMD specialist, with a mean clinical experience of 11.0 ± 5.6 years (range: 6–17). Inter-rater reliability for accuracy ratings was good to excellent (average measures ICC = 0.892, 95% CI: 0.843–0.928) (23).

The patient assessment panel included six adults with confirmed TMD (4 female, 2 male; mean age: 36.2 ± 13.7 years; range: 19–54; median disease duration: 6 weeks, range: 2 weeks–3 months). Health literacy distribution was: low (NVS 0–2), *n* = 2; moderate (NVS 3–4), *n* = 2; high (NVS 5–6), *n* = 2. Each patient evaluated a randomly assigned subset of 20–24 texts. Patient-based findings should be interpreted as exploratory given the modest sample size. PEMAT inter-rater reliability was excellent (ICC(2,1) = 0.941, 95% CI: 0.916–0.959; ICC(2,k) = 0.970, 95% CI: 0.956–0.979).

The characteristics of the Delphi panel, professional assessors, patient assessor were summarized in Supplementary Table S3.

### 3.2 Objective readability (four-group comparison a, B-Claude, C, D)

Objective readability indicators for the 15 texts in each group (A, B-Claude Opus 4.6, C, D) are summarized in Table 1. All parameters differed significantly among groups (all *p* < 0.001).

**Table 1:** Objective readability indicators across the four study groups.

Indicator	Group A (Original)	Group B (Optimal LLM)	Group C (Clinician)	Group D (Human-AI)	*p* value
Mean sentence length	33.9 ± 1.4	21.1 ± 1.3	27.3 ± 2.0	20.4 ± 1.0	< 0.001*
Total character count	2,438 ± 187	4,847 ± 521	5,156 ± 737	6,162 ± 748	
Total sentence count	71 ± 5	220 ± 23	182 ± 23	289 ± 36	
Common character count	1,268 ± 96	1,268 ± 96	2056 ± 283	2,255 ± 274	
Common character proportion (%)	56.9 ± 2.8	48.8 ± 2.4	44.1 ± 2.3	39.9 ± 2.0	< 0.001*
Professional term density (%)	43.1 ± 2.8	51.2 ± 2.4	55.9 ± 3.3	60.1 ± 2.0	< 0.001*

Values are presented as mean ± standard deviation. Statistical details and post-hoc comparisons are reported in the text (Section 3.2). Total character count includes punctuation marks, Arabic numerals, and Latin letters. Common character count refers to the total number of character occurrences (not unique characters) belonging to the Grade-1 Commonly Used Standard Chinese Characters list. Common character proportion = common character count ÷ total character count × 100%. Professional terminology density = 100% − common character proportion.

Mean sentence length differed significantly [*F*(3, 42) = 276.4, *p* < 0.001, η^2^ = 0.952]. Group A had the longest sentences (33.9 ± 1.4 characters), followed by Group C (27.3 ± 2.0), Group B (21.1 ± 1.3), and Group D (20.4 ± 1.0). Post-hoc comparisons (Bonferroni correction) confirmed a graded pattern: A > C > B > D, with A significantly longer than all others (*p* < 0.001), C significantly longer than B and D (*p* < 0.001), and a small but significant difference between B and D (*p* = 0.021).

Total character count differed significantly [*F*(3, 42) = 132.1, *p* < 0.001, η^2^ = 0.904]: Group A (2,438 ± 187) < Group B (4,847 ± 521) < Group C (5,156 ± 737) < Group D (6,162 ± 748). All pairwise differences were significant (*p* < 0.001) except B vs. C (*p* = 0.038). Total sentence count also differed significantly (χ^2^(3) = 42.0, *p* < 0.001, ε^2^ = 0.933): Group D (289 ± 36) > Group B (220 ± 23) > Group C (182 ± 23) > Group A (71 ± 5), with all pairwise differences significant (*p* < 0.001) except B vs. C (*p* = 0.012).

Common character proportion differed significantly [*F*(3, 42) = 153.7, *p* < 0.001, η^2^ = 0.917]: Group A (56.9% ± 2.8%) > Group B (48.8% ± 2.4%) > Group C (44.1% ± 2.3%) > Group D (39.9% ± 2.0%) (all pairwise *p* < 0.05). Professional terminology density showed the inverse pattern [*F*(3, 42) = 153.7, *p* < 0.001, η^2^ = 0.917]. Absolute common character count increased across groups [*F*(3, 42) = 98.3, *p* < 0.001, η^2^ = 0.875]: Group A (1,268 ± 96) < Group B (2,125 ± 229) < Group C (2056 ± 283) < Group D (2,255 ± 274), with all pairwise differences significant (*p* < 0.001) except B vs. C (*p* = 0.045).

### 3.3 Accuracy and completeness (four-group comparison a, B-Claude, C, D)

Inter-rater reliability for accuracy ratings was good to excellent (average measures ICC = 0.892, 95% CI: 0.843–0.928). Accuracy scores differed significantly among the four groups [*F*(3, 42) = 37.8, *p* < 0.001, η^2^ = 0.730; Table 2]. Group C (4.48 ± 0.53) and Group D (4.49 ± 0.36) scored significantly higher than Group A (3.15 ± 0.78) and Group B (3.23 ± 0.69) (all *p* < 0.001). No significant differences were found between C and D (*p* = 1.000) or between A and B (*p* = 1.000).

**Table 2:** Accuracy and completeness scores across the four groups, assessed by the professional panel using a 5-point Likert scale.

Indicator	Group A (Original)	Group B (Optimal LLM)	Group C (Clinician)	Group D (Human-AI)
1. What is TMD	3.0	2.4	4.0	4.4
2. TMD self-screening: When to see a doctor	4.2	4.2	4.6	4.6
3. Diagnostic examinations for TMD	4.0	4.6	4.6	5
4. Disease course and natural history of TMD	4.0	3.4	5.0	4.6
5. Etiology and risk factors of TMD	3.4	3.6	3.8	4.6
6. Conservative treatment modalities and suitable candidates	4.0	4.0	4.6	4.6
7. Is surgery necessary for TMD? Surgical indications	2.6	3.4	5.0	5.0
8. Orthodontic treatment and TMD	2.6	2.8	4.4	4.8
9. Dietary guidance for TMD patients	2.4	3.0	5.0	4.2
10. Stress management and relaxation training	2.4	3.6	4.2	4.0
11. Dos and don’ts in daily life for TMD patients	3.2	3.2	5.0	4.6
12. Acupuncture as an adjunctive treatment for TMD	2.0	2.4	3.8	4.0
13. Common medications for TMD	4.4	4.0	5.0	4.6
14. Prognosis of TMD and integrative Chinese-Western medicine	2.8	2.8	4.0	3.8
15. Occlusal splint therapy: principles, wearing instructions, and precautions	2.6	2.0	3.2	4.2
Accuracy score (1–5)	3.15 ± 0.78	3.23 ± 0.69	4.48 ± 0.53	4.49 ± 0.36

Values represent the mean of five independent expert ratings per text on a 5-point Likert scale (1 = multiple critical errors or omissions, 5 = fully accurate and complete). ICC (average measures, two-way random-effects model, absolute agreement) = 0.892 (95% CI: 0.843–0.928), indicating good to excellent inter-rater reliability. Repeated-measures ANOVA: *F*(3, 42) = 37.8, *p* < 0.001, η^2^ = 0.730. Post-hoc pairwise comparisons with Bonferroni correction: Group C and Group D were each significantly higher than Group A and Group B (all *p* < 0.001). No significant differences were found between Group C and Group D (*p* = 1.000) or between Group A and Group B (*p* = 1.000).

Individual topic scores (Table 2) ranged from 2.0 (Topic 12, acupuncture) to 4.4 (Topic 13, medications) in Group A. TCM-related topics (Topic 12, acupuncture; Topic 14, TCM prognosis) received the lowest scores across all groups (Group C: 3.8; Group D: 4.0). Claude Opus 4.6 was selected as the optimal LLM for Group D and the sensitivity analysis based on accuracy scores.

### 3.4 Actionability (seven-group comparison including all LLM subgroups)

PEMAT inter-rater reliability was excellent (ICC(2,1) = 0.941, 95% CI: 0.916–0.959). Actionability scores differed significantly across all seven groups (Friedman χ^2^(6) = 83.3, *p* < 0.001, Kendall’s W = 0.793; Table 3). Group A scored 16.7% ± 17.6% (0/15 topics ≥ 70%). Group C scored 46.7% ± 20.9% (0/15 ≥ 70%). All four LLM subgroups scored significantly higher than both A and C (all *p* ≤ 0.002): GPT-4 (80.0% ± 22.2%, 7/15 ≥ 70%), Claude Opus 4.6 (95.6% ± 11.3%, 13/15 ≥ 70%), DeepSeek-V2 (97.8% ± 8.6%, 14/15 ≥ 70%), Gemini 3.0 pro (95.6% ± 11.3%, 13/15 ≥ 70%). No significant differences were found among the four LLM subgroups (all *p* ≥ 0.052). Group D scored 95.6% ± 11.3% (13/15 ≥ 70%), significantly higher than A and C (*p* < 0.001) and statistically indistinguishable from all LLM subgroups (*p* = 0.088 to 1.000).

**Table 3:** Comparison of PEMAT actionability scores across study groups.

Group	PEMAT score (%)	Topics≥70%, *n* (%)
Group A (original)	16.7 ± 17.6	0/15 (0%)
Group C (clinician)	46.7 ± 20.9	0/15 (0%)
Group B (GPT-4)	80.0 ± 22.2	7/15 (46.7%)
Group B (Claude Opus4.6)	95.6 ± 11.3	13/15 (86.7%)
Group B (Deepseek-V2)	97.8 ± 8.6	14/15 (93.3%)
Group B (Gemini 3.0 pro)	95.6 ± 11.3	13/15 (86.7%)
Group D (human-AI)	95.6 ± 11.3	13/15 (86.7%)

PEMAT, Patient Education Materials Assessment Tool (actionability subscale). Values are presented as mean ± standard deviation. Scores ≥ 70% indicate acceptable actionability per the PEMAT guidelines. Inter-rater reliability (ICC, two-way random-effects model, absolute agreement): ICC(2,1) = 0.941 (95% CI: 0.916–0.959); ICC(2,k) = 0.970 (95% CI: 0.956–0.979), indicating excellent reliability. Omnibus Friedman test: χ^2^(6) = 83.3, *p* < 0.001, Kendall’s W = 0.793. Post-hoc comparisons were performed using Dunn’s test with Bonferroni correction (adjusted α = 0.00238). Group A was significantly lower than all other groups (all *p* ≤ 0.006). Group C was significantly lower than all LLM groups and Group D (all *p* ≤ 0.002). No significant differences were found among Group D and the four LLM subgroups (all *p* ≥ 0.052).

### 3.5 Exploratory prompt sensitivity analysis (three prompt conditions vs. group C)

Accuracy scores showed a borderline-significant gradient across the three prompt conditions (Friedman χ^2^(2) = 6.00, *p* = 0.050): Prompt A (2.53 ± 0.61), Prompt B (2.67 ± 0.46), Prompt C (2.80 ± 0.40). Only Prompt C vs. Prompt A reached significance (*p* = 0.046, Bonferroni-corrected). All prompt conditions scored below Group C (4.00 ± 0.92). Topic 12 (acupuncture) consistently received the lowest accuracy (2.4 across all prompts), while Topic 11 (dos and don’ts) remained stable at 3.2. Results above were shown in Supplementary Table S4.

### 3.6 Cultural adaptability

Qualitative thematic analysis of all 90 texts identified four themes (Table 4). LLM texts used culturally appropriate institution names (“口腔科,” “中医院”) and direct address (“你”), whereas clinician texts used formal third-person (“患者”). LLM dietary and lifestyle recommendations were structured as itemized behavioral lists with Chinese household food examples; clinician texts embedded guidance within continuous prose. LLM texts employed engagement features (metaphors, “
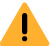
” markers), while clinician texts maintained a neutral professional register. For TCM safety content, LLM texts provided explicit, itemized warnings; clinician texts conveyed equivalent information in narrative paragraphs. Group D preserved LLM structural features while incorporating clinician refinements.

**Table 4:** Summary of qualitative thematic analysis on cultural adaptability.

Theme	Core finding	Illustrative examples
Terminology localization	LLM-generated texts used culturally appropriate Chinese healthcare institution names (e.g., “口腔科,” “口腔颌面外科,” “中医院,” “针灸科”) and addressed patients using “你” (you) rather than “患者” (patient). TCM terminology was generally rendered with conceptual accuracy, though with less pathophysiological depth than clinician texts.	Group B: “请到有资质的中医院或综合医院的中医科/针灸科就诊” (Visit a qualified TCM hospital or the TCM/acupuncture department of a general hospital). Group C: “针灸作为中医特色疗法，具有调和气血、疏通经络、缓急止痛的作用” (Acupuncture as a distinctive TCM therapy harmonizes qi and blood, dredges meridians, and relieves pain).
Dietary and lifestyle recommendation concordance	LLM-generated texts provided dietary and lifestyle recommendations that were generally aligned with Chinese household practices. Occasional Western-default tendencies in earlier model outputs were corrected in the final LLM version. Clinician texts offered more condensed dietary guidance integrated within broader self-management instructions.	Group B: Dietary recommendations used Chinese household food examples and were presented as structured behavioral lists (see this table). Group C: Dietary guidance was embedded within dense paragraphs without itemized food examples.
The culture register of empathy	LLM-generated texts employed emotionally supportive language and used visual emphasis markers (e.g., “ 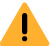 ”) to highlight safety-critical information. Clinician texts maintained a neutral, information-dense professional register throughout.	Group B: “您可以把它想象成关节的’减震垫’——就像跑步时穿的减震鞋垫一样” (You can think of it as a ‘shock absorber’ for your joint—just like the cushioning insoles worn when running). Group C: “咬合板治疗是TMD最重要的保守治疗方式之一” (Occlusal splint therapy is one of the most important conservative treatments for TMD).
TCM content safety and specificity	LLM-generated texts provided explicit, itemized safety warnings with specific clinical scenarios for TCM procedures. Clinician texts presented TCM safety information in narrative paragraphs with comparable medical accuracy but less behavioral specificity.	Group B (acupuncture): Five numbered safety reminders covering facility qualification, self-treatment prohibition, medical condition disclosure, adjunctive role, and interprofessional coordination (see this table). Group C (acupuncture): Single narrative sentence listing contraindicated conditions.

TCM, Traditional Chinese Medicine. Themes were derived from thematic framework analysis of all 90 texts (Group A: 15 original texts; Group B: 15 LLM-rewritten texts by Claude Opus 4.6; Group C: 15 clinician-rewritten texts; Group D: 15 human-AI collaboration texts). Two researchers independently coded a 20% sample of texts; inter-coder agreement exceeded 80%. Representative excerpts were translated from the original Chinese by the research team. Illustrative examples represent the predominant contrast pattern (Group B vs Group C) identified across all four themes; Group D texts, produced through human-AI collaboration, generally preserved the LLM’s structural and engagement features while incorporating clinician refinements.

### 3.7 Efficiency assessment

LLM generation required < 10 s per text (< 3 min for 15 topics). Clinician *de novo* rewriting (Group C) required 18.5 ± 4.2 min per text (≈ 4.6 h total). Clinician review of LLM drafts (Group D) required 5.1 ± 1.8 min per text (≈ 1.3 h total), representing a 72% time saving compared with Group C (Supplementary Table S5).

## 4 Discussion

### 4.1 Summary of principal findings

This study provides the first comprehensive, multi-dimensional evaluation of LLM performance in optimizing Chinese-language PEMs for TMD. Our findings reveal a consistent pattern: LLMs excel in structural and behavioral dimensions—producing texts with substantially shorter sentences and dramatically higher actionability—while expert clinicians remain indispensable for ensuring medical accuracy and cultural appropriateness. The human-AI collaboration model preserved the LLM’s advantages in syntactic simplification and behavioral instruction while elevating accuracy to a level equivalent to clinician-only texts, with a 72% reduction in clinician time investment.

### 4.2 Readability: syntactic simplification and the paradox of lexical dilution

The LLM’s significant reduction in sentence length is consistent with evidence from English-language studies across multiple specialties (14–16) and extends this finding to the Chinese context, where high character density makes syntactic simplification particularly valuable.

A counterintuitive finding was that common character proportion decreased from original to LLM-rewritten texts, despite the latter being judged easier to understand. This paradox is explained by structural expansion: total character count increased by 99% as technical terms were unpacked into explanatory phrases. The absolute number of common characters increased (from 1,268 to 2,125 occurrences), but the denominator grew faster, producing a lower proportion. We term this “structural expansion with lexical dilution”—it reflects the greater text volume required for accessibility, not increased vocabulary difficulty. Researchers evaluating Chinese text simplification should therefore interpret character-frequency indicators alongside absolute counts and sentence length, rather than in isolation.

Clinician-rewritten texts showed only modest syntactic simplification, consistent with evidence that healthcare professionals overestimate patient health literacy and default to professional register (6, 24). This underscores the potential value of LLMs as tools to bridge this persistent gap.

### 4.3 Accuracy: the indispensability of expert review

The LLM preserved but did not enhance source content accuracy, and its accuracy gaps were systematic, clustering in domains requiring precise procedural knowledge and TCM representation. TCM-related topics received the lowest scores across all groups, consistent with evidence that LLM performance degrades for domain-specific knowledge underrepresented in training data (25, 26).

This domain-dependent pattern aligns with findings from other specialties. Karagöz et al. reported variable LLM accuracy on the AAOS Orthopaedic In-Training Examination, with performance consistently below thresholds for unsupervised clinical application (18), and demonstrated that model accuracy in septic arthritis management varied substantially across clinical scenarios requiring integration of multiple findings and procedural decision-making (27). This convergence suggests LLM performance in patient education is systematically influenced by domain representation in training data and reliance on explicit versus experience-based reasoning.

Critically, the human-AI collaboration model remediated these accuracy deficits, achieving accuracy equivalent to clinician-only texts. This provides empirical support for the collaborative workflow proposed by Ali and colleagues (19) and extends its validation to the Chinese-language TMD context.

### 4.4 Actionability: the most clinically consequential finding

Across 30 human-written texts, not a single one met the PEMAT acceptability threshold, while LLM-generated texts achieved acceptable actionability for the majority of topics. This carries particular weight for TMD, where successful treatment depends on consistent patient self-management behaviors. The qualitative case comparison (Table 5) illuminates the mechanism: clinician texts used principle-based recommendations that are medically accurate but behaviorally underspecified, consistent with experts’ tendency to overestimate patients’ procedural knowledge (28). The LLM, by defaulting to discrete, sequenced, and verifiable actions, addresses this gap directly.

**Table 5:** Representative case comparison of actionability: text excerpts illustrating the contrast between clinician and LLM outputs.

Topic	Group	Text excerpt	PEMAT item coverage
Acupuncture for TMD: safety precautions	Group C (Clinician)	“It is worth noting that not all patients are suitable candidates for acupuncture. Patients who are pregnant (especially in early pregnancy), have bleeding disorders, are taking anticoagulant medications, have severe heart disease, have a history of fainting during acupuncture, or have local skin infections should inform their physician to rule out contraindications.”	✓ Item 1 (identifies action: inform physician) Item 2 (third-person phrasing; does not use “you”) Item 3 (lists contraindications as narrative paragraph, not as explicit steps) Item 4 (no safety boundary beyond listing conditions) N/A Items 5–7
	Group B (Claude Opus 4.6)	“ 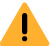 Important safety reminders: 1. Must be performed at a licensed medical facility: Visit a qualified TCM hospital or the acupuncture department of a general hospital. Ensure the procedure is performed by a licensed TCM practitioner. 2. Do not attempt self-acupuncture: Acupoint selection requires professional judgment. Improper needling can cause harm. 3. Actively inform your physician of your medical conditions: If you have a bleeding disorder, are taking anticoagulant medications, are pregnant, or have a skin infection, be sure to inform the acupuncturist in advance. 4. Acupuncture is an adjunctive therapy: It should be used in combination with other treatments, not as a replacement for all treatment. 5. Better results when combined with Western medical treatment: It is recommended to incorporate acupuncture into your overall treatment plan, with coordinated care between your dental and TCM providers.”	✓ Item 1 (identifies 5 specific actions) ✓ Item 2 (uses “you” and “your” throughout) ✓ Item 3 (5 numbered, explicit steps with rationale) ✓ Item 4 (safety warnings with specific clinical scenarios) N/A Items 5–7
Occlusal splint cleaning and maintenance	Group C (Clinician)	“During the wearing process, patients should try to find the mandibular rest position, reduce the frequency of clenching on the splint, and consume a soft diet. The splint and oral cavity must be cleaned promptly after eating to maintain good hygiene. The overall treatment course is approximately 3–6 months. Patients require regular follow-up visits and must not continue wearing the splint long-term without adjustment, as this may lead to secondary occlusal problems.”	✓ Item 1 (identifies actions: find rest position, reduce clenching, soft diet, clean, follow up) Item 2 (uses “patients” rather than “you”) Item 3 (multiple actions compressed into dense paragraph without stepwise decomposition) Item 4 (no specific safety boundaries, durations, or warning signs) N/A Items 5–7
	Group B (Claude Opus 4.6)	“For daily cleaning: 1. After removing the splint each day, gently brush it under running water using a soft-bristled toothbrush. 2. Clean with cool or lukewarm water (Important: Never use hot water! Hot water will cause deformation). 3. When not wearing, store the splint in a clean, dedicated case and keep it ventilated and dry. 4. Before each use, check that the splint has not deformed or become damaged. 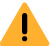 Special precautions: • Do not continue wearing the splint long-term without follow-up visits—prolonged use without adjustment may cause tooth movement or occlusal changes. • If you feel pain or excessive pressure on any tooth while wearing, contact your doctor promptly. • If the splint develops cracks or shows significant wear, have it replaced promptly. • The splint is part of a comprehensive treatment plan and should be used together with other approaches (such as relaxation training and dietary adjustments) for optimal results.”	✓ Item 1 (identifies multiple specific actions) ✓ Item 2 (uses “you” and “your” throughout) ✓ Item 3 (numbered steps for cleaning + bulleted precautions with specific scenarios) ✓ Item 4 (explicit safety warnings: no hot water with rationale, signs requiring dental contact, replacement triggers) N/A Items 5–7

PEMAT, Patient Education Materials Assessment Tool (actionability subscale). ✓ = item adequately addressed in the excerpt;  = item not adequately addressed; N/A = item not applicable to this material type (Items 5–7 pertain to calculations, charts, and visual aids, which are not applicable to text-only patient education materials). Excerpts were selected from the two topics showing the largest qualitative contrast in behavioral instruction specificity between Group C (Clinician) and Group B (Claude Opus 4.6).

An important methodological consideration is the non-equivalence of task instructions. The LLM received explicit actionability-oriented guidance, whereas clinicians wrote under real-world conditions. The higher LLM scores should therefore be interpreted as demonstrating reliable meeting of actionability standards when appropriately prompted, rather than inherent superiority. The prompt sensitivity analysis confirmed that this advantage is partly contingent on behavioral instruction requirements.

The human-AI collaboration model maintained actionability equivalent to the best-performing LLMs, demonstrating that clinicians preserved the LLM’s structural advantages. The consistency across all four LLM subgroups suggests this is a shared property of current-generation models, consistent with recent comparative studies (17). That experienced clinicians collectively failed to meet the PEMAT threshold argues for systematically incorporating tools that bridge the gap between clinical expertise and patient-facing behavioral communication.

### 4.5 Cultural adaptability: beyond translation to cultural localization

The LLM was effective at operational cultural localization—terminology, dietary recommendations, and safety information structure—but less developed at the conceptual level, particularly for TCM content. A noteworthy finding pertained to the cultural register of empathy: LLM texts employed direct address, emotionally supportive framing, and everyday metaphors rarely seen in Chinese clinical documentation, which maintained a neutral professional register. This divergence highlights that conventions of written patient education may differ from those of clinical documentation. Whether Chinese patients prefer the more engaged LLM style or the more reserved clinician style warrants dedicated research.

The dissociation between behavioral explicitness and medical accuracy for TCM content reinforces the indispensability of expert review for materials involving traditional medicine. The human-AI collaboration model offers a practical framework: the LLM supplies patient-facing accessibility and behavioral structure, while the clinician provides domain-specific conceptual enrichment and cultural attunement.

### 4.6 Clinical implementation

The 72% reduction in clinician time transforms patient education material production from a prohibitive burden into a feasible task. Based on our findings, we propose a structured review checklist: (1) verify medical accuracy, especially medication details, procedural precautions, and TCM-specific safety warnings; (2) check cultural concordance of dietary, lifestyle, and healthcare references; (3) assess empathy register alignment with local patient communication norms; (4) verify completeness of safety warnings, particularly TCM contraindications; (5) preserve the stepwise, actionable structure of the LLM draft. Implementing this workflow requires institutional investment in LLM platform access, staff training, and quality monitoring—investments modest relative to the benefit of providing patients with materials that are simultaneously accurate, readable, and actionable.

### 4.7 Limitations

Several limitations should be acknowledged. The 15 topics, while systematically selected through a Delphi process, do not exhaust all TMD educational needs. The patient sample was modest (*n* = 6) and single-center; patient-based findings should be considered exploratory. The study evaluated static materials rather than clinical outcomes, which requires future RCT evaluation. LLM technology is evolving rapidly, and findings represent a current snapshot. TCM content evaluation was limited to practitioners from a single institution.

The LLM and clinician groups received different task instructions: the LLM prompt explicitly requested features aligning with PEMAT actionability criteria, whereas clinicians were instructed to produce optimal materials reflecting real-world practice. This non-equivalence may have contributed to the observed actionability gap. The sensitivity analysis partially mitigated this concern by confirming the advantage was contingent on behavioral instruction requirements. The clustering of LLM and human-AI texts at the upper PEMAT scale may reflect a ceiling effect, and the PEMAT assesses text-level features rather than behavioral outcomes. The sensitivity analysis, limited to three topics, should be considered exploratory. The prompts differed not only in tone but also in content and instruction intensity. Claude Opus 4.6 was selected post-hoc, which may limit generalizability; future studies should prespecify the collaboration model. Efficiency data relied on self-reported clinician times, and the 72% time saving should be interpreted as an estimate rather than a precise measurement.

Generalizability is constrained linguistically, clinically and contextually. Specifically speaking, our findings are specific to Chinese, a language with unique features including high character density and compound terminology formation (10). LLM performance patterns may differ for languages with different orthographic, syntactic, or lexical characteristics. TMD is a condition heavily dependent on patient self-management behaviors, which may make actionability particularly important. The actionability deficit we observed in human-written texts may be less pronounced for conditions where patient education primarily conveys factual knowledge rather than behavioral instructions. The integration of TCM content in our materials reflects the Chinese healthcare context, where TCM is integrated into mainstream care. The cultural adaptability findings may not directly translate to healthcare systems without comparable traditional medicine integration.

### 4.8 Future directions

The next step is an RCT comparing clinical outcomes between TMD patients receiving standard verbal education, clinician-written PEMs, and human-AI collaboration PEMs. The evaluation framework should be applied to other specialties to determine whether the actionability deficit and LLM workflow benefits generalize. Replication in other languages and healthcare contexts is needed to establish cross-linguistic validity. Cross-cultural comparative studies would be valuable given our finding that cultural adaptation requires attention beyond linguistic translation. The review checklist warrants prospective evaluation in clinical settings.

## 5 Conclusion

Under the conditions of this study, the human-AI collaboration model—in which LLMs generate structurally optimized drafts and expert clinicians review for accuracy and cultural appropriateness—represents a promising and balanced approach for producing Chinese TMD patient education materials. Large language models demonstrate advantages in syntactic simplification and behavioral instruction, while expert review remains essential for ensuring medical accuracy and cultural safety, particularly for TCM content. The substantial reduction in clinician time achieved by the collaborative model addresses a key barrier to providing high-quality written patient education. These findings underscore that the responsible deployment of AI in patient education requires cultural adaptation as a core requirement, not an afterthought.

## Data Availability

The original contributions presented in the study are included in the article/Supplementary material, further inquiries can be directed to the corresponding author.
