# Supplementary material for: Large language models versus expert clinicians in optimizing Chinese patient education materials for temporomandibular disorders: a mixed-methods study integrating readability, accuracy, actionability, and cultural adaptability
Source: Front Public Health. 2026 Aug 4;14:1923536. doi: 10.3389/fpubh.2026.1923536 (PMC13481503; doi:10.3389/fpubh.2026.1923536)
Supplement: Supplementary file 1 [file Supplementary_file_1.docx]

**Checklist:**

**第一部分：35个主题的标准答案清单要点**

这是专业评估组评分的核心依据。每个主题列出必须覆盖的关键知识点，评估者据此用1-5分李克特量表评分。

**板块一：疾病认知与基础概念**

**主题1：什么是颞下颌关节紊乱病（TMD）？**

- 定义：TMD是一组累及颞下颌关节、咀嚼肌及相关结构的功能紊乱性疾病的总称，不是单一疾病。
- 主要症状三大类：①疼痛（关节区、面部、耳前、咀嚼肌）；②关节杂音（弹响、摩擦音、捻发音）；③下颌运动异常（张口受限、偏斜、卡顿/锁住）。
- 强调其为常见病、多发病，患病率约5%-15%，可发生于任何年龄，以青壮年女性多见。
- 非致命性疾病，不直接威胁生命，但慢性疼痛可显著影响生活质量（进食、言语、睡眠、情绪）。
- 属于口腔颌面外科/口腔科/颞下颌关节专科的诊疗范围。
- 中医视角可补充：属于中医“颌痹”“颊车骱痛”“口噤”等范畴。

**主题2：TMD的常见分类与各类型特点**

- 按DC/TMD分类：①关节源性（关节盘移位、关节炎、关节强直等）；②肌源性（肌筋膜疼痛、肌痉挛等）；③混合型（最常见）。
- 关节盘移位：可复性（弹响为主，张口可复位）与不可复性（张口受限，弹响消失）。
- 骨关节炎/骨关节病：关节区疼痛伴骨性结构改变，中老年多见。
- 肌筋膜疼痛：咀嚼肌压痛、僵硬、牵涉痛，常与压力相关。
- 强调多数患者为轻度、可逆性，不必然进展为严重阶段。

**主题3：TMD的常见症状：疼痛、弹响、张口受限**

- 疼痛：最常见主诉，可为钝痛、酸痛、刺痛，位于耳前、颞部、下颌角、咀嚼肌群；可放射至头、颈、肩。
- 弹响/杂音：关节盘复位时产生“咔嗒”声；骨性摩擦音提示关节面退变。
- 张口受限：正常张口度约35-50mm（三横指）；轻度受限25-35mm，中度15-25mm，重度<15mm。
- 伴随症状：耳鸣、耳闷胀感、头晕、牙齿咬合不适、面部肌肉疲劳。
- 症状常有波动性，与情绪、压力、疲劳明显相关。

**主题4：TMD的流行病学：发病率、好发人群、性别差异**

- 患病率：一般人群中TMD症状发生率约5%-15%，需要治疗者约3%-7%。
- 年龄高峰：20-45岁青壮年。
- 性别差异：女性显著多于男性，男女比例约1:2至1:6，可能与激素、疼痛敏感性及心理社会因素有关。
- 危险人群：高压力职业者、学生、有焦虑/抑郁倾向者、有口腔不良习惯者。

**主题5：【新增】TMD的病程特点与自然发展史：它会越来越严重吗？**

- 大多数TMD呈波动性、间歇性病程，而非持续恶化。
- 很多患者症状可自行缓解或经保守治疗后显著改善。
- 少数患者（约5%-10%）可能发展为慢性持续性疼痛，需要多学科干预。
- 关键信息：TMD不是“不治之症”，也不是“必然会恶化”的退行性疾病。
- 早期干预、纠正不良习惯、管理压力，是阻断慢性化的关键。

**主题6：【中医】中医如何认识TMD？——从“颌痹”“颊车骱痛”说起**

- 中医病名归属：“颌痹”“颊车骱痛”“口噤”“牙关不利”等。
- 基本病机：局部经筋损伤、气血运行不畅，与肝、脾、肾三脏功能失调相关。
- 常见证型：①风寒湿痹阻（外邪侵袭，关节冷痛，遇寒加重）；②气滞血瘀（外伤或劳损，刺痛固定）；③肝郁气滞（情志不畅，胀痛随情绪波动）；④肝肾不足（久病体虚，关节松弛无力）。
- 中医治疗原则：舒筋通络、调和气血、疏肝解郁、补益肝肾，强调辨证论治。
- 与现代医学的融合：中医从整体调理出发，与现代医学的局部精准诊治互为补充。

**板块二：病因与危险因素**

**主题7：TMD的常见病因与危险因素：多因素致病模型**

- 核心概念：TMD是多因素共同作用的结果，非单一病因。
- 五大类因素：①解剖/咬合因素（非唯一原因，仅起部分作用）；②心理社会因素（压力、焦虑、抑郁）；③不良习惯（紧咬牙、夜磨牙、咬物癖）；④创伤（急性大张口损伤、头颈部挥鞭伤）；⑤全身因素（遗传、激素、关节韧带松弛）。
- 强调：多数因素是可干预、可管理的，患者有主动参与康复的空间。

**主题8：心理压力、焦虑与TMD的关系**

- 心理压力是TMD最重要的诱发和加重因素之一。
- 机制：压力→咀嚼肌紧张度↑（潜意识紧咬牙）→肌肉疲劳/痉挛→疼痛→更多压力（恶性循环）。
- 焦虑/抑郁与TMD慢性疼痛高度共病，DC/TMD Axis II专门评估心理社会状态。
- 缓解建议：腹式呼吸、正念减压、适度运动，必要时寻求心理专业帮助。

**主题9：不良口腔习惯与TMD：紧咬牙、夜磨牙、咬颊咬唇**

- 白天紧咬牙：常不自觉地发生，尤其在专注、紧张、操作精细动作时。
- 夜间磨牙/紧咬：睡眠中发生，可能本人不知，同床者告知或牙医检查发现牙齿磨耗。
- 其他不良习惯：咬唇、咬颊、咬指甲、咬笔杆、嚼口香糖过度。
- 行为干预：自我觉察+“嘴唇合拢、牙齿分开”的姿势训练。
- 治疗：咬合板保护牙齿、缓解肌肉紧张；生物反馈训练。

**主题10：【新增】TMD与磨牙症的关系及管理**

- 磨牙症定义：睡眠或清醒状态下不自主的咀嚼肌节律性活动，分为睡眠磨牙症和清醒磨牙症。
- 与TMD关系：磨牙症是TMD的重要危险因素，尤其在肌源性TMD中。但并非所有磨牙者都会发展为TMD。
- 诊断：病史+临床检查（牙齿磨耗、咬肌肥大、舌缘齿痕）+ 多导睡眠监测（确诊睡眠磨牙症）。
- 管理原则：①咬合板（保护牙齿，减轻肌肉负荷）；②行为干预（生物反馈、自我觉察）；③压力管理；④药物（肌松药、肉毒素注射，需在专科医生指导下进行）。
- 误区纠正：咬合板不能“治愈”磨牙症，主要起保护作用。

**主题11：【新增】颞下颌关节的解剖结构：一张图看懂你的关节**

- 位置：位于耳前，连接下颌骨与颅骨的关节。
- 核心结构：①关节窝（颅骨上的凹陷）；②髁突（下颌骨的圆头）；③关节盘（夹在中间的软骨垫，起缓冲和协调运动作用）；④关节囊和韧带（包裹和稳定关节）。
- 功能：人体最复杂的关节之一，同时完成转动（开口初期）和滑动（开口后期）两个动作。
- 通俗比喻：像一个“带软垫的铰链+滑轨”，关节盘就是那个软垫。
- 了解结构的意义：理解弹响来自关节盘移位，摩擦音来自关节盘磨损或骨骼直接接触。

**板块三：诊断与检查**

**主题12：怀疑TMD时需要做哪些检查？**

- 第一步：详细问诊（症状、病程、诱因、既往治疗）。
- 第二步：临床检查（触诊关节和咀嚼肌有无压痛、听诊有无杂音、测量张口度及运动轨迹、咬合检查）。
- 第三步：影像学检查按需选择：
  - 全景片/曲面断层片：初筛，看骨性结构大体轮廓。
  - CBCT（锥形束CT）：详细评估骨性结构（髁突形态、骨质改变、关节间隙）。
  - MRI（磁共振）：评估软组织的金标准，看关节盘位置、形态、有无穿孔、关节腔积液。
- 强调：检查是根据病情分级选择的，并非“越贵越好”，不是所有患者都需要做MRI。

**主题13：TMD的影像学检查：X线、CBCT、MRI各自的用途**

- 全景片：筛查用，辐射低、费用低；只能看骨的大体轮廓，不能看软组织。
- CBCT：三维看骨性结构（髁突骨质、关节间隙），辐射量高于全景片但远低于医学CT；适合怀疑骨关节病、髁突吸收等。
- MRI：无辐射，软组织成像金标准；清晰显示关节盘位置（正常/可复性前移/不可复性前移）、关节盘形态、关节腔积液、骨髓水肿；是诊断关节盘移位的首选方法。
- 检查流程说明：MRI检查时间较长（约20-30分钟），需保持不动，有幽闭恐惧者可提前与医生沟通。

**主题14：【新增】TMD的自我筛查：我该什么时候去看医生？**

- 建议就诊的信号（出现以下任一情况）：
  - 反复或持续的颌面部疼痛，尤其是耳前、脸颊、太阳穴区域。
  - 张口困难或咀嚼时疼痛。
  - 关节有明显弹响且伴有疼痛或张口受限。
  - 下颌卡住或锁住。
  - 上述症状持续超过2周未缓解。
  - 伴随头痛、耳鸣且已排除其他专科问题。
- 不建议自行诊断或随便找人“正骨复位”，需由口腔颌面外科/TMD专科医生明确诊断。
- 就诊前准备：记录症状出现的时间、诱因、疼痛评分、已尝试的缓解方法。

**主题15：【新增】DC/TMD诊断标准简介：医生是如何诊断TMD的？**

- DC/TMD是目前国际最公认的TMD诊断分类标准（2014年发布）。
- 结构：Axis I（躯体诊断）+ Axis II（心理社会评估）。
- Axis I：通过标准化的问诊和临床检查流程，将TMD分为关节痛、肌痛、关节盘移位、关节炎等具体诊断。
- Axis II：评估疼痛相关功能受限、心理困扰、焦虑、抑郁等，因为这些因素显著影响治疗效果。
- 患者须知：医生问很多问题（包括情绪、压力、睡眠）是因为TMD与心理状态高度相关，不是“怀疑你在想太多”，而是科学评估的必要组成部分。

**板块四：治疗方法与选择**

**主题16：TMD有哪些治疗方法？一张清单看懂全部选择**

- 核心传递“阶梯式治疗”理念：从无创到有创，从保守到手术。
- 第一阶梯（基础/首选）：健康教育、行为纠正、自我护理。
- 第二阶梯（保守治疗）：物理治疗（理疗/手法）、咬合板、药物、心理干预。
- 第三阶梯（微创操作）：关节腔穿刺冲洗、关节腔注射（透明质酸/激素）、关节镜手术。
- 第四阶梯（开放手术）：关节盘复位术、关节成形术等（极少用，<5%患者需要）。
- 强调：绝大多数患者在前两阶梯即可获得满意改善，微创和手术仅适用于经规范保守治疗无效的少数病例。

**主题17：TMD的保守治疗方式详解及适合人群**

- 保守治疗范畴：健康教育、行为纠正、物理治疗、咬合板、药物。
- 物理治疗：热敷/冷敷、超声波、经皮电刺激（TENS）、低能量激光、咀嚼肌按摩、下颌运动训练。
- 行为纠正：软食、避免大张口、戒除紧咬牙/咬物习惯、打哈欠时用手托下巴。
- 心理干预：认知行为疗法、放松训练、生物反馈。
- 适合人群：绝大多数TMD患者，尤其是初次诊断、症状轻中度、肌源性为主的患者。
- 保守治疗的核心优势：无创、可逆、成本低、患者可主动参与。

**主题18：TMD一定要做手术吗？什么情况才需要考虑手术？**

- 明确回答：绝大多数（>90%-95%）TMD患者不需要手术。
- 手术的严格指征（需同时满足）：①明确的结构性病变（如关节盘不可复性前移伴严重机械性障碍、骨性强直、关节肿瘤）；②经正规、充分的保守治疗（≥3-6个月）效果不佳；③疼痛或功能障碍严重影响生活质量。
- 手术种类简述：关节镜（微创，恢复快）、开放关节手术（创伤大，恢复期长）、全关节置换（终末期选择，极少用）。
- 强调：手术不是根治手段，术后仍需配合保守康复和自我管理。
- 如何判断：由TMD专科医生综合评估后，与患者共同决策。

**主题19：咬合板治疗须知：原理、佩戴方法与注意事项**

- 原理：①提供稳定的咬合接触，减少咀嚼肌异常活动；②保护牙齿免受磨耗；③在部分患者中可引导关节盘复位。
- 种类：稳定型咬合板（最常用）、再定位咬合板、软弹性咬合板（短期用）。
- 佩戴方法：通常夜间佩戴，部分患者根据医嘱日间也需佩戴。
- 初期适应：异物感、唾液增多是正常反应，通常1-2周内逐渐适应。晨起咬合不适感也常见。
- 清洁护理：牙刷+清水刷洗，可用假牙清洁片浸泡，不可用热水（会变形）。
- 复诊调整：需要多次复诊调磨，确保咬合均匀。不可自行停戴或修改。
- 疗程：通常数月至一两年，需根据病情个体化。

**主题20：【新增】物理治疗在TMD中的应用：超声波、激光、手法治疗**

- 物理治疗是保守治疗的重要组成部分，通常由康复科/理疗科医生或物理治疗师执行。
- 常用方法：
  - 超声波治疗：深部组织加热，缓解肌肉痉挛和疼痛，促进局部血液循环。
  - 低能量激光：减轻关节炎症反应、镇痛、促进组织修复。
  - 经皮电刺激（TENS）：通过电流刺激减轻疼痛和肌肉紧张。
  - 手法治疗：专业治疗师对紧张的咀嚼肌和颈部肌肉进行松解，改善关节活动度。
- 治疗频次：通常每周2-3次，疗程遵医嘱。
- 强调：物理治疗需在专业人员指导下进行，患者不可自行对关节施力“正骨”。

**主题21：【新增】TMD的微创治疗：关节腔穿刺、灌洗与关节镜手术**

- 关节腔穿刺冲洗：用生理盐水冲洗关节上腔，清除炎性介质和微小粘连，缓解疼痛和改善活动度。
- 关节腔注射：在冲洗后可注射透明质酸钠（润滑和保护关节软骨）或少量激素（短期强力抗炎）。
- 关节镜手术：通过微小切口置入内窥镜，可在直视下松解粘连、复位关节盘、清理炎性组织。创伤小，恢复较快。
- 适应症：经保守治疗3-6个月效果不佳的中度关节源性TMD（关节盘移位伴疼痛、关节炎早期）。
- 术后须知：软食1-2周，配合康复训练，循序渐进恢复功能。

**主题22：【新增】正畸治疗与TMD的关系：矫牙能治疗TMD吗？**

- 核心结论：正畸治疗不是TMD的常规治疗方法。
- 目前科学证据：正畸治疗不能可靠地“治愈”或“预防”TMD；TMD也不是正畸治疗的禁忌症。
- 咬合与TMD的关系：咬合因素在TMD发病中仅起次要作用，咬合不良不等同于TMD。
- 正畸中的注意事项：如果正畸期间出现TMD症状，应及时告知正畸医生，可能需要暂停加力，对症处理。
- 正畸后：稳定的咬合关系可能对部分患者有间接益处，但不应以此作为治疗TMD的主要目的。
- 患者须知：如果有TMD症状且有正畸需求，应在TMD症状稳定控制后再开始正畸，并由口腔正畸科和TMD专科医生协作管理。

**板块五：自我管理与康复**

**主题23：TMD患者的居家物理治疗与自我保健**

- 热敷：适用于慢性期或晨起肌肉僵硬。用40-45℃温热毛巾或热水袋包裹毛巾，敷于疼痛侧耳前、面颊，每次15-20分钟，每天2-3次。
- 冷敷：适用于急性发作期或术后24-48小时内。用毛巾包裹冰袋，每次10-15分钟，间隔至少2小时。
- 咀嚼肌按摩：以指腹轻柔打圈按摩咬肌和颞肌，避开痛点用力按压；每次5-10分钟，力度以感到舒适放松为宜。
- 下颌运动训练：在无痛范围内，面对镜子缓慢匀速练习张口-闭口、下颌左右偏、前伸运动，纠正偏斜。每次练习5-10次，每天2-3组。
- 强调：所有操作应在医生指导下进行，如疼痛加剧应停止并及时复诊。

**主题24：TMD患者的饮食指导：吃什么与不吃什么**

- 核心原则：选软不选硬，选小不选大，选温不选冷。
- 推荐食物：粥、蒸蛋羹、豆腐、炖烂的肉/鱼、蔬果泥/汁、酸奶、面条。
- 需避免/限制：坚果、牛肉干、甘蔗、法棍等硬质面包、嚼口香糖、啃骨头、啃苹果（切小块可）、冰水/冰品。
- 进食方式：小口慢嚼，双侧交替咀嚼，避免单侧咀嚼加重一侧关节负担。
- 中医疗法补充：避免过食生冷（伤阳气）、辛辣（助火生热）、发物（可能加重炎症反应），具体遵中医师指导。

**主题25：TMD患者的咀嚼肌自我按摩与放松技巧**

- 咬肌按摩：双手食指、中指、无名指并拢，以指腹在面颊两侧咬肌区域由下向上、由浅入深轻柔打圈。
- 颞肌按摩：用指尖在太阳穴附近及耳廓上方区域轻柔打圈。
- 翼内肌/翼外肌的间接放松：张口2cm，轻轻左右摆动下颌，感受肌肉的拉伸与放松。
- 放松时刻：热水淋浴时、睡前、长时间说话或咀嚼后。
- 技巧要点：力度宁轻勿重，找到酸胀但不痛的力度；疼痛明显时暂停按摩，改热敷。

**主题26：【新增】TMD患者的姿势纠正与人体工学建议**

- 头前伸姿势与TMD：长期低头（看手机、电脑）导致颈后肌群紧张、下颌骨被动后下移位，增加颞下颌关节和咀嚼肌负荷。
- 改善建议：
  - 坐姿：臀坐满椅子，腰背挺直有支撑，屏幕高度与视线平齐或稍低，双肩放松下沉。
  - 手机使用：将手机举至与视线平齐，避免长时间低头。
  - 睡姿：避免俯卧（头颈扭转压迫关节），建议侧卧或仰卧，枕头高度适中，能填充头颈与床面的空隙。
  - 工作中：每45-60分钟起身活动，做颈部和肩部的拉伸。
- 简单自查：靠墙站立，后脑勺、肩胛骨、臀部、脚跟贴墙，感受头颈中立位。

**主题27：【新增】TMD与睡眠质量的关系及改善建议**

- 双向关系：疼痛影响睡眠，睡眠差加重疼痛（恶性循环）。
- TMD患者常见睡眠问题：入睡困难（疼痛干扰）、睡眠维持困难（夜磨牙/紧咬牙）、晨起时颌面部僵硬酸痛。
- 改善建议：
  - 睡姿：侧卧或仰卧，避免俯卧压迫面部和关节。
  - 睡前1小时：避免使用电子屏幕；温水淋浴或泡脚；腹式呼吸放松。
  - 疼痛管理：睡前遵医嘱使用止痛药或进行热敷。
  - 磨牙干预：佩戴咬合板。
- 强调：长期睡眠障碍需排查其他原因（如睡眠呼吸暂停），必要时做多导睡眠监测。

**主题28：【新增】TMD患者的压力管理与放松训练**

- 压力与TMD的直接关联：压力→肌肉紧张→疼痛→更多压力→慢性化。
- 腹式呼吸法（简便有效的即时放松）：鼻吸4秒→屏息2秒→口呼6秒，感受腹部起伏而非胸部。每次5分钟，每日多次，睡前必做。
- 渐进式肌肉放松：依次收紧→放松身体各肌群（脚→小腿→大腿→腹→胸→肩→颈→面），感受紧张与放松的对比。
- 正念练习：将注意力放在当下呼吸和身体感受上，不评判。推荐手机App辅助。
- 生活方式：规律作息、适度有氧运动（步行、瑜伽、太极）、维持社交、减少咖啡因摄入。
- 专业支持：若自我调节效果有限，可寻求心理咨询/认知行为疗法。

**主题29：【新增】TMD患者的下颌运动训练：怎么做才安全？**

- 目的：改善张口度和下颌运动的对称性，预防关节僵硬。
- 准备：面对镜子，放松肩颈。
- 动作一（张口-闭口练习）：舌尖轻抵上腭前部（控制运动轨迹），缓慢张口至无痛最大范围，保持3秒，再缓慢闭口。全程观察下巴是否偏向一侧，努力保持中线。重复5-10次/组，每天2-3组。
- 动作二（左右偏练习）：牙齿轻微分开，缓慢将下颌向左移动至无痛范围，保持3秒，回中位；再向右移动。重复5-10次/侧。
- 动作三（前伸练习）：牙齿轻微分开，缓慢将下颌向前伸出（地包天姿势），至无痛范围，保持3秒，回中位。重复5-10次。
- 安全原则：全程在无痛范围内进行（如有疼痛立即减小幅度或停止）；动作匀速、缓慢；任何新训练开始前需经医生或治疗师确认。

**主题30：【新增】TMD患者日常生活的“该做与不该做”**

- 该做：
  - 吃软食，切小块，双侧咀嚼。
  - 打哈欠时用手托住下巴。
  - 保持“嘴唇合拢、牙齿分开”的休息位（上下牙之间保持2-3mm缝隙）。
  - 规律进行医生指导下的康复训练。
  - 天冷时注意颌面部保暖，可戴口罩或围巾。
- 不该做：
  - 避免大张口（打哈欠、咬大苹果、看牙时及时提醒医生）。
  - 避免嚼口香糖、啃硬物、用牙开瓶盖。
  - 避免长时间单侧咀嚼或趴睡。
  - 避免刻意反复去听关节弹响。
  - 避免听信非专业人士的“正骨复位”。

**板块六：药物、中医与整合管理**

**主题31：TMD常用药物须知：止痛药、肌松药与抗炎药**

- 药物是辅助治疗，不是核心治疗，需配合行为纠正和物理治疗。
- 非甾体抗炎药：如布洛芬、双氯芬酸钠。作用：抗炎镇痛。注意：饭后服用减少胃肠刺激；有胃溃疡、肾病、心血管疾病史者需告知医生。
- 肌松药：如盐酸乙哌立松。作用：缓解咀嚼肌痉挛和紧张。副作用：可能有嗜睡、乏力，服药期间避免驾车和操作精密机械。
- 三环类抗抑郁药（低剂量）：如阿米替林。作用：慢性疼痛的神经调节，非主要抗抑郁用途。副作用：口干、便秘、嗜睡，通常睡前服用。
- 用药原则：遵医嘱按时按量，不可自行增减剂量或停药；定期复诊评估疗效和副作用。
- 中医中药：需由中医师辨证处方，不可自行购买或照方抓药。

**主题32：【中医】针灸如何辅助治疗TMD？**

- 原理：调和气血、疏通经络、缓急止痛；现代研究显示针灸可促进内源性阿片肽释放、改善局部血液循环。
- 常用穴位：下关、颊车、听宫、翳风、合谷、足三里、太冲等（根据辨证加减）。
- 治疗过程：使用一次性无菌针具，针刺特定穴位，留针15-30分钟；可能配合电针增强刺激。
- 针感说明：酸、麻、胀、重是正常得气感，非疼痛。
- 治疗频次：急性期可每周3-5次，稳定期每周1-2次，疗程遵医嘱。
- 安全性：必须由持证中医师操作；针具严格无菌一次性使用。
- 不适用/需告知中医师的情况：怀孕（尤其早期）、出血性疾病、正在服用抗凝药物、严重心脏病、晕针史、局部皮肤感染。

**主题33：【中医】如何使用中药草药包进行局部热敷？**

- 原理：温经通络、散寒止痛、舒筋活血。适用于虚寒型、气滞血瘀型TMD。
- 草药包来源：由中医师辨证开具处方，通常包含艾叶、伸筋草、透骨草、红花等。
- 操作步骤（逐条细化）：
  1. 将草药包用清水浸湿（约1分钟）。
  2. 放入蒸锅或微波炉加热至温热潮湿（非滚烫）。
  3. 取出后，用干毛巾包裹草药包（至少2-3层）。
  4. 温度测试：将包裹好的草药包贴于手腕内侧皮肤，感觉温热而不烫（约40-45℃）。
  5. 敷于疼痛侧耳前、面颊、下颌角区域，每次15-20分钟，每天1-2次。
  6. 热敷后立即用干毛巾擦干局部，避风寒，30分钟内不外出。
- 安全警告（重点强调）：
  1. 不可过热，谨防烫伤。
  2. 皮肤有破损、皮疹、感染者禁用。
  3. 急性红肿热痛期（属热证、炎症明显）不宜用热敷，需遵医嘱。
  4. 孕妇腹部和腰骶部禁用，面部热敷需中医师确认。
- 需在中医师指导下使用，不可自行随意配伍草药。

**主题34：【中医】口服中药治疗TMD的须知与饮食禁忌**

- 核心强调：必须由中医师辨证论治后开具处方，不可自行照方抓药或服用他人药方。
- 常见治法方向（仅供参考，不作处方依据）：疏肝解郁（如逍遥散加减）、活血化瘀（如身痛逐瘀汤加减）、祛风散寒除湿（如蠲痹汤加减）、补益肝肾（如独活寄生汤加减）。
- 煎煮方法（一般常规）：
  - 将一剂中药放入砂锅/不锈钢锅，加冷水没过药面约2-3cm，浸泡30分钟。
  - 武火（大火）煮沸后，转文火（小火）煎煮20-30分钟，倒出药液。
  - 同上法加温水再煎一次，将两次药液混合，分早晚两次服用。
  - 特殊煎法（先煎、后下、包煎、烊化等）遵医嘱。
- 服用时间：一般饭后半小时温服，减少胃肠刺激。
- 饮食禁忌（核心文化内容）：
  - 忌生冷：冰饮、生食、凉拌菜。
  - 忌辛辣：辣椒、花椒、生姜（除外作为药引时）、大蒜。
  - 忌油腻：油炸食品、肥肉。
  - 忌发物：海鲜（虾蟹贝类）、羊肉、鹅肉、韭菜、香菇（具体遵医嘱，不同证型禁忌不同）。
- 与西药间隔至少1小时服用，并告知中西医双方所有正在使用的药物。

**主题35：TMD的预后与中西医协同管理的展望**

- 总体预后：良好。绝大多数TMD患者可通过保守治疗获得症状显著改善或完全消失。
- TMD是“可管理”的慢性问题：类似“关节的感冒”，诱因出现时可能复发，但可通过自我管理控制发作频率和程度。
- 自我管理的核心地位：习惯纠正、压力管理、康复训练是防止复发的基础。
- 中西医各自优势定位：
  - 西医：在精准诊断（影像学、DC/TMD分型）、结构修复（手术、关节镜）、急性炎症控制上有不可替代的优势。
  - 中医：在缓解慢性疼痛、改善肌肉功能、调理体质、减少复发、提高生活质量上有独特价值，尤其适合对西药不耐受或偏好自然疗法的患者。
- 理想模式：中西医协同，由口腔科/TMD专科医生主导诊断和主体治疗方案，由中医师辅助进行体质调理和疼痛管理。双方信息互通，避免药物冲突。
- 强调：中西医协同不等于“替代”，不可因偏信中医而拒绝必要的西医诊疗。
