# Supplementary material for: Large language models versus expert clinicians in optimizing Chinese patient education materials for temporomandibular disorders: a mixed-methods study integrating readability, accuracy, actionability, and cultural adaptability
Source: Front Public Health. 2026 Aug 4;14:1923536. doi: 10.3389/fpubh.2026.1923536 (PMC13481503; doi:10.3389/fpubh.2026.1923536)
Supplement: Supplementary file 3 [file Table_1.docx]

**15 topics**

| Topic number | English | Chinese |
| --- | --- | --- |
| 1 | What is Temporomandibular Disorders (TMD)? | 什么是颞下颌关节紊乱病（TMD）？ |
| 2 | Self-Screening for TMD: When Should I See a Doctor? | TMD的自我筛查：我该什么时候去看医生？ |
| 3 | What Examinations Are Needed if TMD Is Suspected? | 怀疑TMD时需要做哪些检查？ |
| 4 | Course and Natural History of TMD: Will It Get Worse? | TMD的病程特点与自然发展史：它会越来越严重吗？ |
| 5 | Common Etiology and Risk Factors for TMD: A Multifactorial Pathogenesis Model | TMD的常见病因与危险因素：多因素致病模型 |
| 6 | Conservative Treatment Modalities for TMD and Suitable Populations | TMD的保守治疗方式详解及适合人群 |
| 7 | Is Surgery Necessary for TMD? Under What Circumstances Should It Be Considered? | TMD一定要做手术吗？什么情况才需要考虑手术？ |
| 8 | The Relationship Between Orthodontic Treatment and TMD: Can Orthodontics Treat TMD? | 正畸治疗与TMD的关系：矫牙能治疗TMD吗？ |
| 9 | Dietary Guidance for TMD Patients: What to Eat and Avoid | TMD患者的饮食指导：吃什么与不吃什么 |
| 10 | Stress Management and Relaxation Training for TMD Patients | TMD患者的压力管理与放松训练 |
| 11 | Dos and Don'ts in Daily Life for TMD Patients | TMD患者日常生活的“该做与不该做” |
| 12 | How Can Acupuncture Assist in TMD Treatment? | 针灸如何辅助治疗TMD？ |
| 13 | Medication for TMD: Analgesics, Muscle Relaxants, and Anti-Inflammatories | TMD常用药物须知：止痛药、肌松药与抗炎药 |
| 14 | Prognosis of TMD and the Prospect of Integrated Chinese-Western Management | TMD的预后与中西医协同管理的展望 |
| 15 | Essentials of Occlusal Splint Therapy: Principles, Wearing Methods, and Precautions | 咬合板治疗须知：原理、佩戴方法与注意事项 |
