# Supplementary material for: Large language models versus expert clinicians in optimizing Chinese patient education materials for temporomandibular disorders: a mixed-methods study integrating readability, accuracy, actionability, and cultural adaptability
Source: Front Public Health. 2026 Aug 4;14:1923536. doi: 10.3389/fpubh.2026.1923536 (PMC13481503; doi:10.3389/fpubh.2026.1923536)
Supplement: Supplementary file 4 [file Table_3.docx]

**Supplementary Table S3 Characteristics of the Delphi panel, professional assessors, and patient assessors.**

| Characteristic | Delphi panel (n=5) | Professional assessors (n=3) | Patient assessor (n=6) |
| --- | --- | --- | --- |
| Age（years）mean±SD | 41.8±7.3 | 39.0±4.6 | 36.2±13.7 |
| Female,n(%) | 4（80.0%） | 1（33.3%） | 4（66.7%） |
| Clinical experience(years),mean±SD | 16.2±8.6 | 11.0±5.6 | —— |
| TMD disease duration，median（range） | —— | —— | 6 weeks（2 weeks-3months） |
| Health literacy level（NVS），n | | | |
| Low（0-2） | —— | —— | 2 |
| Moderate（3-4） | —— | —— | 2 |
| High（5-6） | —— | —— | 2 |
| Professional background，n | | | |
| TMD specialist | 1 | 1 | —— |
| Oral and maxillofacial surgeon | 1 | 1 | —— |
| General dental practitioner | 1 | 1 | —— |
| Rehabilitation specialist | 1 | —— | —— |
| Nursing/patient education | 1 | —— | —— |

*Note.* NVS = Newest Vital Sign (Chinese version). TMD disease duration was calculated from the date of initial clinical diagnosis to the date of study participation. The Delphi panel, professional assessors, and text-generating clinicians were mutually exclusive groups with no overlapping membership.
