# Supplementary material for: Large language models versus expert clinicians in optimizing Chinese patient education materials for temporomandibular disorders: a mixed-methods study integrating readability, accuracy, actionability, and cultural adaptability
Source: Front Public Health. 2026 Aug 4;14:1923536. doi: 10.3389/fpubh.2026.1923536 (PMC13481503; doi:10.3389/fpubh.2026.1923536)
Supplement: Supplementary file 5 [file Table_4.docx]

**Supplementary Table S4.** **Prompt sensitivity analysis: PEMAT actionability and accuracy scores under three prompt conditions compared with clinician-written texts.**

| Dimension and topic | Prompt A（Empathetic） | Prompt B（Concise） | Prompt C （Authoritative） | Group C （Clinicians） |
| --- | --- | --- | --- | --- |
| **PEMAT action ability（%）** | | | | |
| Topic 11 Dos and don’ts | 100 | 66.7 | 100 | 66.7 |
| Topic12. Acupuncture | 100 | 33.3 | 100 | 33.3 |
| Topic 15. Occlusal splint | 100 | 66.7 | 100 | 33.3 |
| Mean | 100 | 55.6 | 100 | 44.4 |
| **Accuracy (1-5)** | | | | |
| Topic 11 Dos and don’ts | 3.2 | 3.2 | 3.2 | 5.0 |
| Topic12. Acupuncture | 2.4 | 2.4 | 2.4 | 3.8 |
| Topic 15. Occlusal splint | 2.0 | 2.4 | 2.8 | 3.2 |
| Mean±SD | 2.53±0.61 | 2.67±0.46 | 2.80±0.40 | 4.00±0.92 |

*Note.* PEMAT = Patient Education Materials Assessment Tool (actionability subscale). Prompt A = original empathetic prompt (identical to main experiment prompt). Prompt B = concise and objective prompt with emotional, cultural, and TCM-specific instructions removed. Prompt C = authoritative professional prompt adopting the persona of a senior oral and maxillofacial surgeon. The full text of all prompts is provided in Supplementary Appendix B. Group C values represent the clinician-written texts for the same three topics from the main experiment (see Table 3). PEMAT scoring: inter-rater reliability ICC(2,1) = 0.941 (95% CI: 0.916–0.959). Accuracy scoring: inter-rater reliability ICC(2,k) = 0.915 (95% CI: 0.696–0.977). Given the small number of topics (n = 3 per condition), descriptive comparisons are presented for PEMAT; no formal statistical testing was conducted. For accuracy scores, Friedman test: χ²(2) = 6.00, P = 0.050, Kendall's W = 1.000. Post-hoc Dunn's test with Bonferroni correction (adjusted α = 0.0167): Prompt C vs Prompt A, P = 0.046; Prompt C vs Prompt B, P = 0.317; Prompt B vs Prompt A, P = 0.317. Statistical comparison between prompt conditions and Group C was not performed due to the small topic sample size.
