# Supplementary material for: Large language models versus expert clinicians in optimizing Chinese patient education materials for temporomandibular disorders: a mixed-methods study integrating readability, accuracy, actionability, and cultural adaptability
Source: Front Public Health. 2026 Aug 4;14:1923536. doi: 10.3389/fpubh.2026.1923536 (PMC13481503; doi:10.3389/fpubh.2026.1923536)
Supplement: Supplementary file 6 [file Table_5.docx]

**Supplementary Table S5.** **Time efficiency comparison across text generation methods.**

| Method | Time per text | Total time for 15 texts |
| --- | --- | --- |
| LLM generation （Claude Opus 4.6） | ＜10 seconds | ＜3 minutes |
| Clinician de novo rewriting（Group C） | 18.5±4.2 minutes | 4.6 hours |
| Clinician review of LLM draft（Group D） | 5.1±1.8 minutes | 1.3 hours |
| Time saved | —— | 72% |

*Note.* LLM generation time represents the duration from prompt submission to output completion, recorded automatically. Clinician time for Group C and Group D was self-reported by the two clinicians who produced these texts.
